# Effects of hypobaric hypoxia and voluntary hypocapnic hyperventilation on metabolic response during high-intensity intermittent exercise

**DOI:** 10.1186/2046-7648-4-S1-A44

**Published:** 2015-09-14

**Authors:** Kohei Dobashi, Kazuhito Watanabe, Bun Tsuji, Yosuke Sasaki, Tomomi Fujimoto, Takeshi Nishiyasu

**Affiliations:** 1 School of Health and Physical Education, University of Tsukuba, Tsukuba, Japan; 2 Institute of Health and Sport Sciences, University of Tsukuba, Tsukuba, Japan; 3 Faculty of Human Development, Kobe University, Kobe, Japan; 4 Graduate School of Comprehensive Human Sciences, University of Tsukuba, Tsukuba, Japan

## Introduction

To improve performance of short time high-intensity exercise, it is important to enhance anaerobic energy systems. High intensity intermittent exercise training stresses the anaerobic energy systems in order for improvements to occur. In addition, it is suggested that high intensity intermittent exercise under hypobaric hypoxia (HX) as well as reductions in arterial CO_2 _pressure (hypocapnia: HC) induced by voluntary hyperventilation could enhance anaerobic metabolic rate more effectively [1]. However, it is unclear whether the effects on anaerobic metabolic rate differ between HX and HC. Therefore, the purpose of this study was to investigate whether HX and HC have similar effects on metabolic response during high intensity intermittent exercise.

## Methods

Ten male athletes (8 sprinters and 2 decathletes) performed high-intensity intermittent cycling exercise consisting of three exercise bouts interspaced with 4 min recovery periods [first (Ex.1) and second (Ex.2) exercise: 30-s high-intensity constant work load exercise {574 (17) W}; third exercise (Ex.3): 30-s maximal exercise]. The exercise was performed under normobaric normoxia (CON), HX (HX; equivalent to 2,500 m above sea level at 560 mmHg and exposure duration was through the experiment or, about an hour), and normobaric normoxia but the subjects performed voluntary hyperventilation before the exercise (minute ventilation of 30 L.min^−1^, 20 min) and the recovery periods (minute ventilation of 40 L.min^−1^, 4 min)(HC). Include the variables measured.

## Results

In Ex.1, O_2 _uptake (VO_2_) in HC was lower than in CON {826(107) vs. 1645 (126) mL.min^−1^}. Also, Blood Lactate concentration (BLa) after Ex.1 was higher in HC than in CON {5.46 (0.1) vs. 4.54 (0.2) mmol.L^−1^}. On the other hand, no differences in VO_2 _and BLa between CON and HX were observed through Ex.1 to Ex.3.

## Discussion

These results suggest that HC induced by voluntary hyperventilation attenuated aerobic energy supply with concomitant increase in anaerobic energy release during Ex.1. In Ex.2 and Ex.3, however, VO_2 _and BLa did not differ between CON and HC. Because reductions in end-tidal CO_2 _pressure (an index of arterial CO_2 _pressure) in HC observed immediately before each exercise were reduced with repeated exercise, the extent of the reduction in arterial CO_2 _pressure might not be enough to induce remarkable changes in metabolic responses in Ex.2 and Ex.3. In addition, acute hypoxic exposures did not affect anaerobic metabolism. This may be due to the short exercise time and the relatively long recovery time in the protocol, this may lead to aerobic recovery occurring in the hypoxic environment. Consequently the anaerobic components of metabolism were not stressed to promote increases in anaerobic metabolism.

## Conclusion

Our findings suggest that, during high-intensity intermittent exercise consisting of three short time (30-s) exercises, HC induced by voluntary hyperventilation reduces aerobic energy supply and enhances anaerobic metabolic rate especially at the first exercise and acute HX (2,500 m above sea level) has no impact on the metabolic response.

## References

[B1] Fujii N Tsuchiya SI Tsuji B Watanabe K Sasaki Y Nishiyasu T Effect of voluntary hypocapnic hyperventilation on the metabolic response during Wingate anaerobic test Eur J Appl Physiol in press 10.1007/s00421-015-3179-8 25944513

